# Modeling of Complex Interfaces: From Surface Chemistry to Nano Chemistry

**DOI:** 10.3390/nano10030540

**Published:** 2020-03-17

**Authors:** Jelle Vekeman, Frederik Tielens

**Affiliations:** General Chemistry (ALGC), Materials Modelling Group, Vrije Universiteit Brussel (Free University Brussels-VUB), Pleinlaan 2, 1050 Brussel, Belgium; Jelle.Vekeman@vub.be

For a few years now, quantum chemical modeling of materials has experienced a tremendous boost due to the increasing computational power. However, regardless of whether Moore’s law is respected or not, the difficulty of modeling has now shifted to the construction of the model itself. Of course, the accuracy of the calculations can still be improved, but the main chemical properties and their trends are relatively well reproduced today, especially when they are combined with experiments. One can say that density functional theory (DFT) is now at a mature age and that it can be used as a reliable prediction tool in material science applications, although some work can be done on accuracy. Nevertheless, DFT is especially efficient in describing chemical phenomena at the molecular level, whereby the studied systems increase continuously in size and complexity. Indeed, the size of the system is directly related to the computation power, and the complexity is related to the quality of the calculation method and the representation of the chemical environment in the model. It is the latter property that brings the computational chemist’s chemical intuition and general chemistry knowledge at the forefront. In this Special Issue, we wanted to focus on the construction of pertinent models that are able to describe and predict, as accurately as possible with the available computational power, the chemistry of materials.

This Special Issue contains 10 articles on the topic of modeling of complex interfaces whereby the depth of the field is nicely reflected in the wide variety of systems that is investigated in this issue. Indeed, studies are included on titanium dioxide, silicon/silicon dioxide, carbide- and graphene-based systems, sulfides, and metals such as tin and ruthenium.

In a first paper, the role of rutile TiO_2_ surface orientation and the associated surface termination on molecular hydrogen activation is systematically investigated at the DFT level by Wei et al. [1]. More specifically, four different orientations were considered, and the activation barriers for the heterolytic dissociation of H_2_ and the subsequent H transfer from Ti to O were calculated.

The second paper, by Hessou et al. [2], discusses the adsorption of dibenzyl disulfide adsorption in faujasite zeolites, a major cause of copper corrosion in electrical transformer oil. After carefully comparing different MY zeolites at the DFT+D level, they found that CsY, AgY and CuY are the most promising adsorbents. Indeed, these materials exhibit a good compromise between high interaction energies and limited S–S bond activation.

In addition to this, there is a paper describing a study of the thermal boundary characteristics of homo- and heterogeneous, reactive, and nonreactive interfaces between two solids by Heijmans et al. [3]. Via a connection between reactive force field molecular dynamics and phenomenological theory, a continuous temperature profile was found for the homogeneous nonreactive interface, while a temperature jump was found for the heterogeneous nonreactive interface. Furthermore, it was found that the thermal boundary resistance was twice as large for reactive interfaces than for nonreactive interfaces.

This issue also includes an article by Black et al. [4] on the investigation of the impact of cross-polymerization on the frictional properties of chemisorbed alkylsilane monolayers on silica surfaces. Using molecular simulations, it was demonstrated that crosslinking, together with a fraction of chemisorbed chains, affects the monolayer structure slightly without significantly impacting the frictional performance. It was the first time the effect of this specific property was isolated.

A Raman spectroscopy investigation by Chong et al. [5] of the strain relaxation from grain boundaries in epitaxial graphene is also presented, whereby the graphene was grown via chemical vapor deposition in SiC. The study shows that multiple boundary-like effects are present in the graphene film, while compressive strain in the film shifts the 2D-band frequency downwards due to strain relaxation. A detailed analysis of the phenomenon is given.

In second study on graphene by Lee et al. [6], the effect of N- and S-doping on the electronic structure was investigated using DFT. It was found that N-doping gives rise to p-type defects, while N/S-doping leads to n-type defects. It is thus suggested that by varying the S concentration, the material can switch between p-type and n-type behavior. This is interesting as both types of doping are shown to lead to different unique electronic properties.

Lu et al. [7] contributed with a study on a single tunnel field-effect transistor. They boosted the performance of the model device via energy-band engineering of the InAs/Si heterojunction and a novel device structure of the source-pocket concept. The model showed and improved the tunnel-on state current and subthreshold swing. Their protocol shows the ability to study Tunnel field-effect transistors-based circuit simulations in significantly less time than conventional methods, maintaining an acceptable accuracy.

A study towards the mitigation of hydrogen uptake in ruthenium aided by tin is presented by Onwudinanti et al. [8] as to avoid blistering of ruthenium-capped multilayer reflectors. DFT calculations and charge density analyses showed a significant drop in the energy barrier for hydrogen to penetrate the ruthenium surface when a tin atom was present. They show that the main reason is a charge transfer from tin to hydrogen and the ruthenium surface.

Friák et al. [9] studied the structural, thermodynamic, and elastic properties of nanocomposites such as transition-metal disilicides and magnetic phases containing Fe and Al. For both types, they used first-principles electronic structure calculations to study a range of different atom orderings and crystal structures and assess their interface energies and related properties. 

Finally, tensile stress effects on the structure and stability of prototypical covalent and layered materials were studied and presented by Chorfi et al. [10] They used DFT calculations to quantify the response of selected covalent and layered materials and to find the ideal strength along their main crystallographic direction. With some exceptions, it was found that increasing transverse stress leads to a decrease of the critical strength. Furthermore, the calculated stress–strain curves were described by a newly proposed spinodal equation of state that is generally applicable.

With this, we hope that readers interested in nanomaterials will find in this Special Issue a nice overview of the field of modeling of complex interfaces: from surface chemistry to nano chemistry. Furthermore, we hope that the included works may inspire the scientific community to further push the boundaries of these interesting topics.
